# Distribution and Characterization of the Novel Quorum-quenching Enzyme AiiB in *Priestia megaterium* Isolated from a Natural Environment

**DOI:** 10.1264/jsme2.ME25004

**Published:** 2025-07-18

**Authors:** Tomohiro Morohoshi, Waka Arai, Kanna Ueno, Nobutaka Someya

**Affiliations:** 1 Graduate School of Regional Development and Creativity, Utsunomiya University, 7–1–2 Yoto, Utsunomiya, Tochigi 321–8585, Japan; 2 Institute for Plant Protection, National Agriculture and Food Research Organization (NARO), 2–1–18 Kannondai, Tsukuba, Ibaraki 305–8666, Japan

**Keywords:** quorum sensing, quorum quenching, *N*-acylhomoserine lactone, *Priestia megaterium*, lactonase

## Abstract

Many plant pathogenic bacteria regulate the expression of virulence factors via *N*-acylhomoserine lactone (AHL), a quorum-sensing signaling compound. When numerous spore-forming bacteria were isolated from a natural environment, *Priestia megaterium* was the dominant species, and some *P. megaterium* strains exhibited AHL-degrading activity. The results of a HPLC ana­lysis of AHL degradation products demonstrated that *P. megaterium* degraded AHL by AHL lactonase, which hydrolyzes the lactone ring of AHL. The novel AHL lactonase gene, *aiiB*, was found in the whole genome sequence of AHL-degrading *P. megaterium*. The relationship between the presence of *aiiB* and AHL-degrading activity in *P. megaterium* strains revealed that *P. megaterium* may be classified into three AHL degradation groups: Group 1 (with AHL-degrading activity and *aiiB*), Group 2 (with neither AHL-degrading activity nor *aiiB*), and Group 3 (without AHL-degrading activity, but with *aiiB*). A comparative genome ana­lysis suggested that *aiiB* was obtained or missed by a non-transpositional event during the process of evolution in *P. megaterium*. The amino acid sequences of AiiB in Group 1 and 3 strains were almost identical, and *Escherichia coli* harboring *aiiB* from Groups 1 and 3 exhibited high AHL-degrading activity. Although the AHL-degrading activity of Group 3 strains was markedly weaker than that of Group 1 strains, they degraded AHL in a long-term incubation. Based on the present results, Group 1 and 3 strains, the genomes of which contain *aiiB*, may reduce potato maceration activity under the control of AHL-mediated quorum sensing in *P. carotovorum* subsp. *carotovorum* NBRC 12380.

In modern agriculture, chemical pesticides are widely used to control various plant diseases (Bardin *et al.*, 2015). However, the excessive use of chemical pesticides has adverse effects on human health and ecosystems (Nicolopoulou-Stamati *et al.*, 2016). Biological pesticides are living organisms that are used to control plant diseases. Since biological pesticides have a number of advantages, such as a smaller impact on organisms in the environment than chemical pesticides, they have attracted attention in recent years as a useful method for reducing the use of chemical pesticides (Stenberg *et al.*, 2021). The effects of biological pesticides are based on some effective abilities, such as the competitive exclusion of plant pathogens by niche occupation and the production of antimicrobial secondary metabolites of volatile organic compounds (Medina *et al.*, 2017). However, there are few examples of biological pesticides that interrupt interactions between plant pathogens and reduce their pathogenicity.

Quorum sensing is a bacterial cell-cell communication mechanism (Parsek and Greenberg, 2000). Many plant pathogenic bacteria produce *N*-acylhomoserine lactone (AHL), a quorum-sensing signaling compound that regulates the expression of virulence factors (Von Bodman *et al.*, 2003). AHL is synthesized by the AHL synthase LuxI, forms a complex with the AHL receptor protein LuxR above the threshold concentration of AHL, and activates the transcription of virulence genes (Parsek and Greenberg, 2000). The disruption of the AHL synthase gene in plant pathogenic bacteria markedly decreases the expression of virulence factors (Pirhonen *et al.*, 1993). Therefore, the removal of AHLs produced by plant pathogenic bacteria is expected to contribute to reductions in the expression of virulence factors under the control of quorum sensing. Many tech­niques for the inhibition of bacterial quorum sensing have been proposed, and one of the most effective methods is the use of AHL-degrading enzymes (Uroz *et al.*, 2009). A previous study demonstrated that a number of bacteria, fungi, plants, and mammals produced AHL-degrading enzymes, and the inhibition of quorum sensing using AHL-degrading enzymes is known as quorum quenching (Uroz *et al.*, 2009). Two major AHL-degrading enzymes are AHL lactonase and acylase. AHL lactonase hydrolyzes the lactone ring of AHL, and AHL acylase hydrolyzes the amide bond between homoserine lactone and the fatty acid in AHL (Uroz *et al.*, 2009). The transformation of AHL-degrading genes into plant pathogenic bacteria and the co-inoculation of plant pathogenic bacteria with AHL-degrading bacteria may inhibit the quorum sensing of plant pathogenic bacteria, thereby suppressing their pathogenic expression (Morohoshi *et al.*, 2009; Wang *et al.*, 2010). These findings suggest the potential of AHL-degrading bacteria as biocontrol agents for controlling plant pathogenic bacteria.

Bacterial strains belonging to the genus *Bacillus* are known to benefit plants by protecting them from plant pathogens and have the ability to form spores that are suitable for microorganism pesticides (Etesami *et al.*, 2023). The first AHL-degrading microorganism identified was *Bacillus* sp. 240B1, and the AHL lactonase gene *aiiA* was cloned from its genome (Dong *et al.*, 2000). The *aiiA* gene has also been identified in the genome of *Bacillus thuringinensis*, which is widely used as an insecticidal biological pesticide (Dong *et al.*, 2004). Therefore, *Bacillus* species with AHL-degrading activity may be used as biocontrol agents against a wide range of plant diseases. *Priestia megaterium* in the present study was previously named *Bacillus megaterium*, but was recently moved to the new genus *Pristia* (Gupta *et al.*, 2020). A previous study demonstrated that cytochrome P450 from *P. megaterium* oxidized AHL and reduced its quorum-sensing activity (Chowdhary *et al.*, 2007). However, there is no evidence to show that *P. megaterium* possesses AHL degradation enzymes apart from AHL-modifying enzymes, such as cytochrome P450. Therefore, we herein investigated the distribution of *P. megaterium* among spore-forming bacteria isolated from soil, plant leaves, and roots, confirmed the AHL-degrading activity of *P. megaterium* isolates, exami­ned the mechanisms underlying AHL degradation, and demonstrated that it may inhibit the pathogenesis of plant pathogenic bacteria by suppressing quorum sensing.

## Materials and Methods

### Bacterial strains, media, compounds, and growth conditions

*Escherichia coli* DH5α was grown at 37°C in Luria-Bertani (LB) medium. *Chromobacterium violaceum* CV026 (McClean *et al.*, 1997) and VIR07 (Morohoshi *et al.*, 2008a) were used as AHL reporter strains that respond to short- and long-chain AHLs, respectively. *Pectobacterium carotovorum* subsp. *carotovorum* NBRC 12380 was used as the model plant pathogenic bacterium (Morohoshi *et al.*, 2019b). *P. megaterium*, *C. violaceum*, and *P. carotovorum* subsp. *carotovorum* were grown at 30°C in LB medium. A solid bacterial medium was prepared by adding agar to a final concentration of 1.5%. Ampicillin was added as required at a final concentration of 100‍ ‍μg mL^–1^. *N*-hexanoyl-l-homoserine lactone (C6-HSL) and *N*-decanoyl-l-homoserine lactone (C10-HSL) were used as AHL substrates in the present study. The PCR primer sequences used in this study are listed in Table S1.

### Isolation and identification of *P. megaterium* strains from the natural environment

To isolate spore-forming bacteria, 130 soil samples, 50 plant leaves, and 53 plant roots were collected from various locations in Japan. Samples were briefly washed, homogenized without surface sterilization, and suspended in sterilized distilled water. The suspensions obtained were incubated at 80°C for 1‍ ‍h to select thermostable spore-forming bacteria. The suspensions were then sequentially diluted and spread on nutrient broth (NB; Nippon Becton Dickinson) containing 50‍ ‍μg mL^–1^ cycloheximide, and the plates were incubated in the dark at 25°C for 72 h. Thereafter, bacterial colonies were randomly selected from those that grew on the NB agar medium. To identify bacterial species, the partial 16S rRNA gene was amplified using Bacterial 16S rDNA PCR Kit Fast (Takara Bio) and sequenced by a Sanger sequencing service (Takara Bio). The partial 16S rRNA sequence of each strain was compared with that of the *P. megaterium* strain, NBRC 15308^T^. Strains with 16S rRNA sequences that showed more than 99% identity with NBRC 15308^T^ were selected for this experiment. The 722 *P. megaterium* strains used in this study are listed in Table S2.

### Screening for AHL-degrading activity in *P. megaterium* strains

*P. megaterium* strains were inoculated into 4‍ ‍mL of LB liquid medium and incubated at 30°C for 18‍ ‍h with shaking. Full-grown cultures (500‍ ‍μL) were collected in new microtubes, and C6-HSL was added to a final concentration of 20‍ ‍μM. Cultures were incubated at 30°C for 3‍ ‍h with shaking and centrifuged to obtain the supernatant. C6-HSL remaining in the supernatant was detected on LB agar plates containing the AHL reporter strain CV026. Briefly, an overnight culture of CV026 was added to melted LB agar medium and solidified in a Petri dish. Paper discs (diameter of 8‍ ‍mm; Advantec) were placed on LB agar plates containing the AHL reporter strain, and 20‍ ‍μL of the supernatant was applied to each paper disc. The plates were incubated at 30°C overnight, and AHL degradation was confirmed by the disappearance of purple pigment production.

To investigate the time course of C6-HSL degradation by *P. megaterium* strains, full-grown cultures mixed with 20‍ ‍μM C6-HSL were incubated at 30°C with shaking. Aliquots were obtained 3, 6, 9, and 24‍ ‍h later and centrifuged to obtain supernatants. The supernatants were applied to paper discs on LB agar plates containing CV026 and incubated at 30°C overnight. The amount of residual C6-HSL was calculated using equations describing the relationship between the size of the purple zone and the amount of AHL (Morohoshi *et al.*, 2008b). Experiments were repeated at least three times.

### HPLC ana­lysis to detect AHL lactonase activity

A HPLC ana­lysis of AHL degradation products was performed using a previously described method with slight modifications (Wang *et al.*, 2010; Morohoshi *et al.*, 2015). *P. megaterium* cells were collected from the full-grown culture by centrifugation, washed with phosphate-buffered saline (PBS), and resuspended in PBS. The cell suspension (40‍ ‍μL) was mixed with 4‍ ‍μL of 1 M phosphate buffer (pH 7.0), 352‍ ‍μL of distilled water, and 4‍ ‍μL of 100‍ ‍mM C10-HSL. After an incubation at 30°C for 1 h, the reaction solution was mixed with 400‍ ‍μL acetonitrile, vortexed, and centrifuged to remove cells. Lactone ring-hydrolyzed C10-HSL was prepared by incubating C10-HSL in 10‍ ‍mM NaOH at 30°C for 30‍ ‍min. Samples (20‍ ‍μL) were chromatographed on an HPLC system (Jasco) with a UV/VIS detector set at 205‍ ‍nm using a Mightysil RP-18GP column (250×4.6‍ ‍mm, particle diameter of 5‍ ‍μm; Kanto Kagaku). Samples were eluted isocratically with 50% water, 50% acetonitrile, and 0.1% acetic acid at a flow rate of 2‍ ‍mL‍ ‍min^–1^.

### Whole-genome shotgun sequencing and bioinformatics

The genomic DNA of *P. megaterium* strains was extracted using the NucleoSpin Microbial DNA kit (Takara Bio). Library construction and sequencing using the DNBSEQ-G400 platform (MGI Tech) were performed using the commercial services of Bioengineering Lab.. Adapter sequences were trimmed from raw reads using Trimmomatic version 0.39 (Bolger *et al.*, 2014). Adapter-trimmed reads were assembled using SPAdes version 4.0.0 (Bankevich *et al.*, 2012). Assemblies were annotated using the DDBJ Fast Annotation and Submission Tool (DFAST) version 1.3.3, a bacterial genome annotation pipeline (Tanizawa *et al.*, 2018). Briefly, coding sequences (CDS) were predicted using MetaGeneAnnotator version 2008/08/19 (Noguchi *et al.*, 2008). Genes coding for tRNA and rRNA were identified using Aragorn 1.2.38 (Laslett and Canback, 2004) and Barrnap 0.8 (https://github.com/tseemann/barrnap), respectively.

Amino acid sequences were aligned and shaded using ClustalW (Thompson *et al.*, 1994) and GeneDoc software (https://nrbsc.org/gfx/genedoc). A phylogenetic tree was constructed using the neighbor-joining method with ClustalW in MEGA 7 software (Kumar *et al.*, 2016). The Basic Local Alignment Search Tool (BLAST) (Altschul *et al.*, 1990) on the National Center for Biotechnology Information (NCBI) website (https://blast.ncbi.nlm.nih.gov/) was used to perform a homology search. A local BLAST search was conducted using Sequenceserver version 2.0.0 (Priyam *et al.*, 2019). Gene cluster comparison figures were generated using clinker version 0.0.31 (Gilchrist and Chooi, 2021). Average nucleotide identity (ANI) values were calculated using the pairwise calculation method with the OrthoANIu online tool (https://www.ezbiocloud.net/tools/ani) (Yoon *et al.*, 2017). Digital DNA-DNA hybridization (dDDH) values were calculated using formula two of the Genome-to-Genome Distance Calculator 3.0 (https://ggdc.dsmz.de/ggdc.php) (Meier-Kolthoff *et al.*, 2022).

### Identification and cloning of *aiiB* in *P. megaterium* strains

To detect the presence of the *aiiB* gene in the genome of *P. megaterium* strains, the internal region of *aiiB* was amplified using TaKaRa Taq HS Fast Detect (Takara Bio) and the specific primer sets, AiiBp-F/AiiBp-R. PCR was performed using the following cycling parameters: at 94°C for 5‍ ‍s, 55°C for 1‍ ‍s, and 68°C for 4‍ ‍s for 27 cycles. PCR amplification was confirmed by agarose gel electrophoresis. To clone *aiiB* from *P. megaterium* strains, *aiiB* coding regions were amplified using KOD FX Neo DNA polymerase (Toyobo) and the specific primer sets, Pme_aiiB-F2/Pme_aiiB-R2 (for PL125 and PR185) or Pme_aiiB-F1/Pme_aiiB-R1 (for other strains). PCR was performed using the following cycling parameters: at 98°C for 10‍ ‍s, 60°C for 30‍ ‍s, and 68°C for 1‍ ‍min for 27 cycles. PCR products were digested with *Eco*RI/*Bam*HI (for PL125 and PR185) or *Eco*RI/*Pst*I (for other strains), inserted into the same restriction sites in the pUC118 vector, and transformed into *E. coli* DH5α. Full-grown cultures (500‍ ‍μL) of *E. coli* DH5α harboring *aiiB* were mixed with C6-HSL or C10-HSL at a final concentration of 20‍ ‍μM. After an incubation at 30°C for 1‍ ‍h with shaking, remaining AHL was detected on LB agar medium containing CV026 (for C6-HSL) or VIR07 (for C10-HSL), as described above.

### Assay for the attenuation of potato tissue maceration

The potato tissue maceration activity of *P. catorovorum* subsp. *catorovorum* NBRC 12380 was evaluated using a previously described method with slight modifications (Morohoshi *et al.*, 2024). Briefly, NBRC 12380 and *P. megaterium* strains were cultivated at 30°C for 8‍ ‍h until they reached full growth. Full-grown cultures were diluted using PBS to approximately 1×10^7^ CFU mL^–1^ (NBRC 12380) and 2.5×10^8^ CFU mL^–1^ (*P. megaterium*). Ten microliters of the cell suspension was spotted onto potato slices. Potato slices were incubated at 30°C in the dark while retaining moisture. After an incubation for 2 days, the development of maceration was observed.

### Nucleotide sequence accession number

The whole-genome sequences and raw sequencing reads of the nine *P. megaterium* strains were deposited in the DDBJ/ENA/GenBank database and DDBJ Sequence Read Archive (DRA), respectively. Accession numbers are listed in Table S3.

## Results and Discussion

### Abundance of *P. megaterium* among spore-forming bacteria isolated from the natural environment

To isolate spore-forming bacteria, soil, plant leaves, and plant roots were collected from various locations in Japan. Homogenized samples were incubated at 80°C for 1 h, spread onto an NB agar plate, and incubated at 25°C for 72‍ ‍h until colonies formed. Colonies were randomly selected, and 1,300, 500, and 530 colonies were obtained from soil, plant leaves, and plant roots, respectively. Genomic DNA was extracted from all colonies, and partial 16S rRNA gene sequences were elucidated and used to identify bacterial species. More than 99.8% of isolates were classified as members of the family *Bacillaceae* (data not shown). When strains with 16S rRNA sequences showing more than 99% identity with that of *P. megaterium* NBRC 15308^T^ were classified as *P. megaterium*, 346 strains from soil (approximately 27%), 172 from plant leaves (34%), and 204 from plant roots (38%) were classified as *P. megaterium* (Table 1 and S1). The next major group was the *B. cereus* group (approximately 11% of all isolates), which was less than half that of *P. megaterium* (data not shown). These results suggest that *P. megaterium* was the dominant species within spore-forming *Bacillaceae* isolated in the present study.

### Identification and characterization of AHL-degrading *P. megaterium* strains

**Fig. 1.**
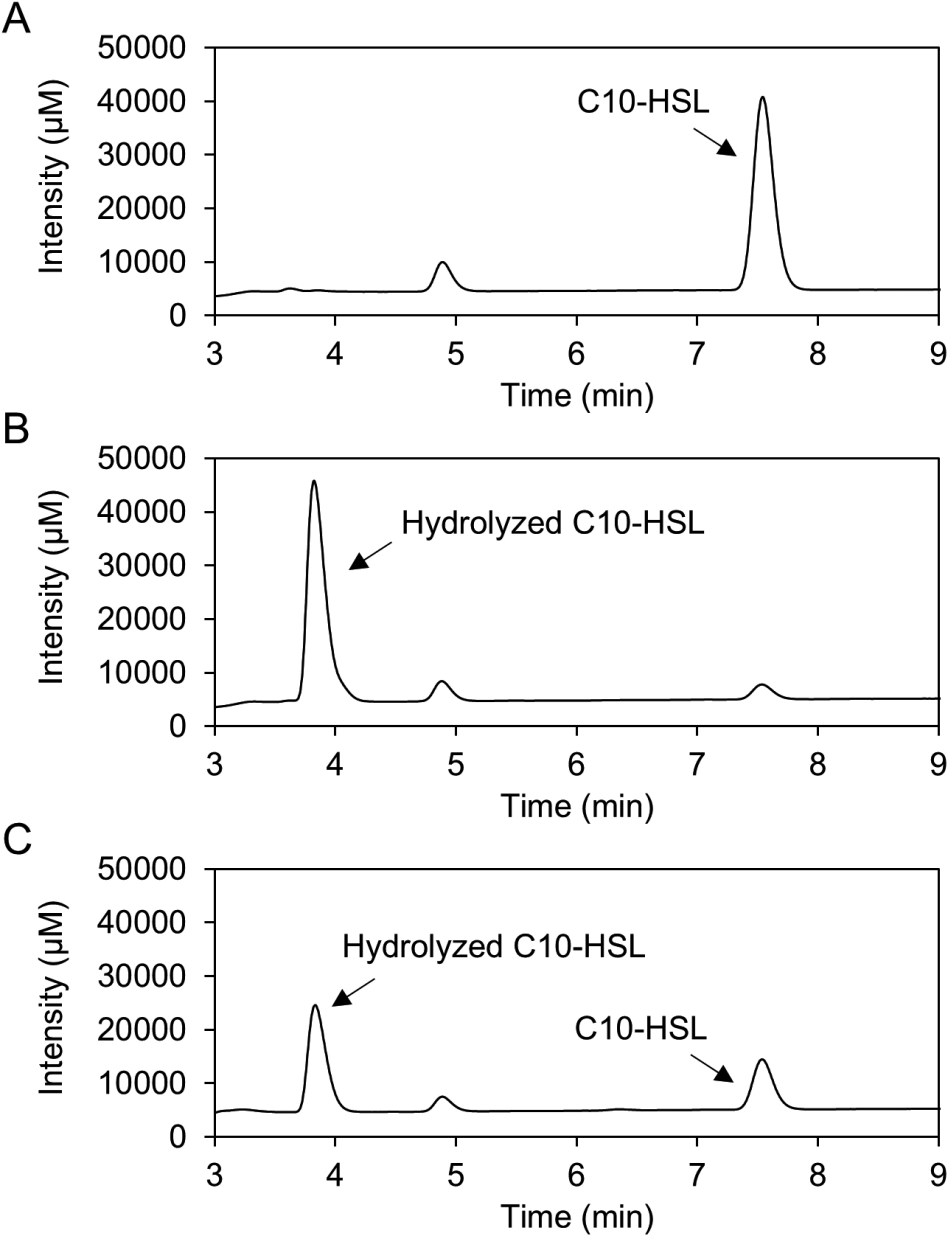
HPLC profiles of C10-HSL (A), lactone ring-hydrolyzed C10-HSL (B), and C10-HSL degraded by *Priestia megaterium* S70. The peaks corresponding to C10-HSL (retention time of approximately 7.5‍ ‍min) and lactone ring-hydrolyzed C10-HSL (3.8‍ ‍min) are indicated by arrows.

To investigate the AHL-degrading activities of *P. megaterium* strains, a full-grown culture of each strain was mixed with 20‍ ‍μM C6-HSL and incubated for 3‍ ‍h with shaking. C6-HSL remaining in the culture supernatant was detected using the AHL reporter strain *C. violaceum* CV026. At this stage, we defined an AHL-degrading *P. megaterium* strain as one that completely degraded 20‍ ‍μM C6-HSL within 3 h. The results obtained are summarized in Table 1, and those for all strains are listed in Table S2. AHL-degrading activity was detected in 58 strains from 346 soil isolates (approximately 17%), 20 from 172 plant leaf isolates (12%), and 19 from 204 soil isolates (9.3%). Although the AHL-degrading activity of *P. megaterium* type strain NBRC 15308^T^ was evaluated, it did not exhibit this activity. These results suggest that AHL-degrading activity was only detected in some *P. megaterium* strains. To investigate the mechanisms by which *P. megaterium* degrade AHL, AHL lactonase activity was investigated in *P. megaterium* strains. The structure of C10-HSL degraded by AHL-degrading *P. megaterium* strain S70 was analyzed by HPLC. Fractionation of the degradation products of C10-HSL revealed two HPLC peaks, which corresponded to those of remaining C10-HSL and lactone ring-hydrolyzed C10-HSL (Fig. 1). These results indicate that *P. megaterium* degraded AHL through its lactonase activity.

### Identification of a novel AHL lactonase gene from the genome sequence of *P. megaterium*

**Fig. 2.**
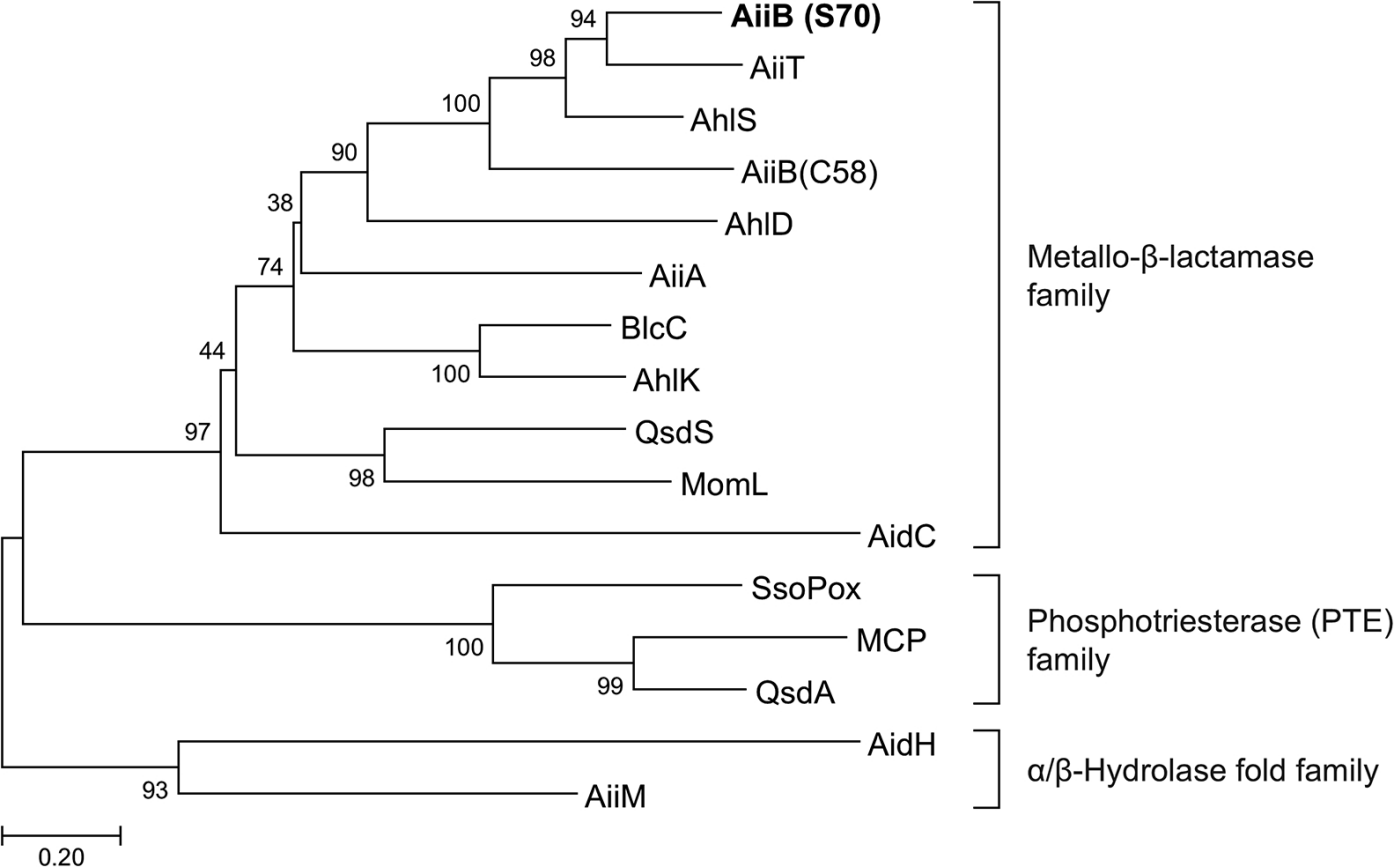
Phylogenetic tree based on amino acid sequences of AiiB from *Priestia megaterium* S70 (bold), AiiB from *Agrobacterium tumefaciens* C58, and other known AHL lactonases belonging to the metallo-β-lactamase family, phosphotriesterase (PTE) family, and α/β-hydrolase fold family. A phylogenetic tree was constructed using the neighbor-joining method with the ClustalW program in MEGA 7 software. The percentage of replicate trees in which the associated taxa were clustered together in the bootstrap test (500 replicates) is shown next to the branches. The scale bar represents 0.2 substitutions per amino acid position.

To identify the presence of an AHL lactonase gene homologue in the genome sequences of AHL-degrading *P. megaterium* strains, whole-genome shotgun sequencing of AHL-degrading *P. megaterium* strain S70 was performed using the DNBSEQ-G400 platform. The general features of the S70 genome are listed in Table S3. Annotation of the S70 genome sequence using the MetaGeneAnnotator program revealed the presence of one CDS (PMEGAS70_21950) assigned to the AHL lactonase gene *aiiB*. The *aiiB* gene has been identified as an AHL lactonase homologue in the genome sequence of *Agrobacterium tumefaciens* C58, and AiiB from C58 exhibits AHL lactonase activity (Carlier *et al.*, 2003). We exami­ned the phylogenetic relationship between the amino acid sequences of AiiB from S70 and known representative AHL lactonases from various bacteria using the neighbor-joining method (Fig. 2). Previous studies reported that the major AHL lactonases belonged to three main protein families: the metallo-β-lactamase family, phosphotriesterase (PTE) family, and α/β-hydrolase fold family (Uroz *et al.*, 2009; Ryu *et al.*, 2020). A phylogenetic ana­lysis indicated that AiiB from S70 belonged to the metallo-β-lactamase family, similar to AiiB from C58. Although AiiB from S70 was phylogenetically close to AiiB from C58, AiiT from *Thermaerobacter marianensis* (Morohoshi *et al.*, 2015) and AhlS from *Solibacillus silvestris* (Morohoshi *et al.*, 2012) were more closely related to AiiB from S70. Since other AHL lactonase and acylase gene homologues were not found in the S70 genome by a local BLAST search (data not shown), AiiB appeared to function as the principal AHL-degrading enzyme in S70.

To investigate the distribution of *aiiB* in the genomes of *P. megaterium* strains, we designed the universal primers AiiBp-F and AiiBp-R to amplify the internal region of *aiiB* and exami­ned the presence of *aiiB* in all *P. megaterium* strains isolated in the present study. The results obtained are summarized in Table 1, and those for all strains are listed in Table S2. The internal region of *aiiB* was successfully amplified from all AHL-degrading *P. megaterium* strains. The internal region of *aiiB* was also amplified in some non-AHL-degrading *P. megaterium* strains, including 25 soil isolates (approximately 8.7%), 17 plant leaf isolates (11%), 26 plant root isolates (14%), and NBRC 15308^T^. Based on these results, we classified *P. megaterium* strains into three AHL degradation groups: Group 1 (with AHL-degrading activity and *aiiB*), Group 2 (with neither AHL-degrading activity nor *aiiB*), and Group 3 (without AHL-degrading activity, but with *aiiB*).

### Comparative genome ana­lysis of selected *P. megaterium* strains

**Fig. 3.**
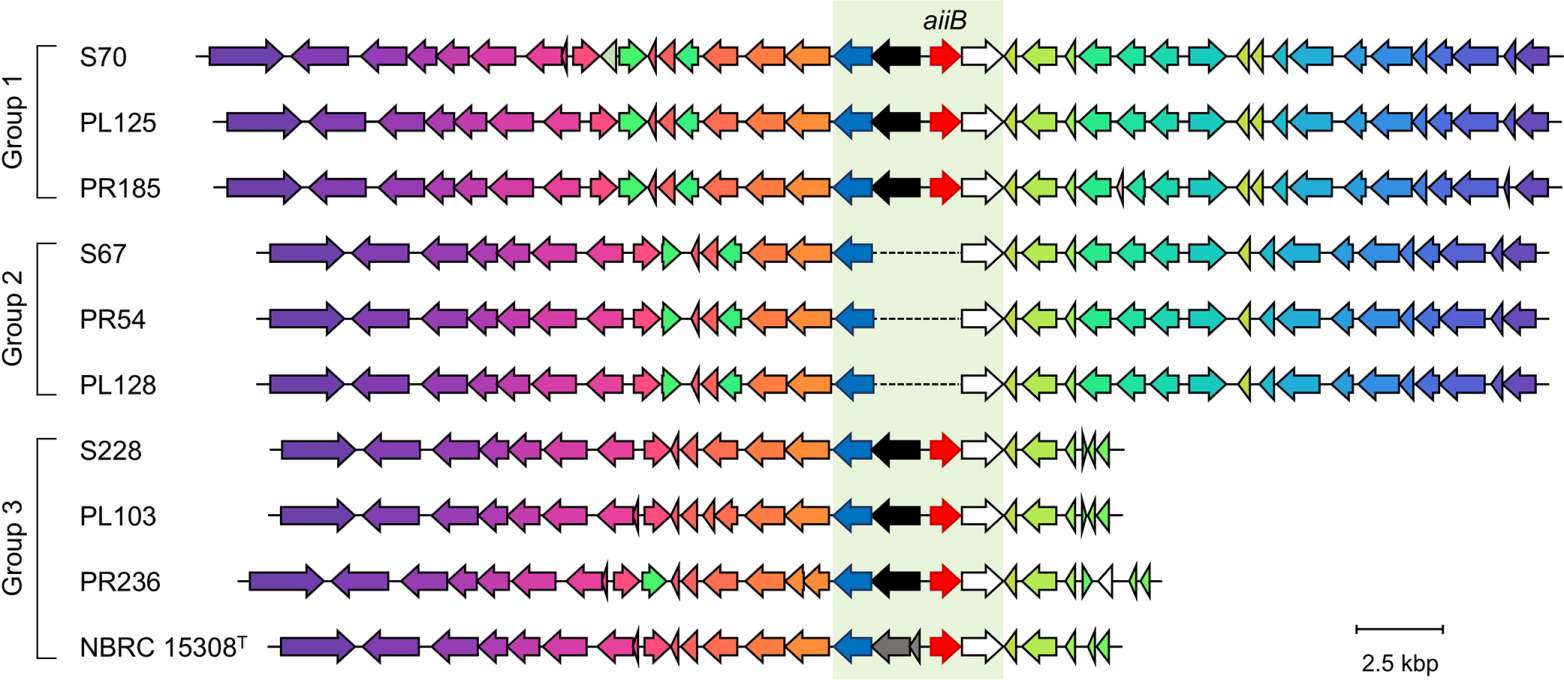
Comparison of *aiiB* and its surrounding genes in genome sequences of *Priestia megaterium* strains. Arrows represent genes, and their direction corresponds to the direction of gene transcription. Genes encoding similar proteins are represented by the same color using the clinker program. The *aiiB* insertion site is shown with a green background. Red, black, white, and blue arrows on the green background indicate *aiiB*, sigma 54-interacting transcriptional regulator, alcohol dehydrogenase, and sugar diacid recognition domain-containing protein genes, respectively. The dark gray arrow indicates an incomplete sigma 54-interacting transcriptional regulator gene.

To identify differences at the genomic level among the three AHL degradation groups, we selected nine *P. megaterium* strains: S70, PL125, and PR185 from Group 1; S67, PL128, and PR54 from Group 2; and S228, PL103, and PR236 from Group 3. Whole-genome shotgun sequencing of these strains was performed using the DNBSEQ-G400 platform. The general features of the nine genomes are listed in Table S3. To evaluate genomic similarities between these nine strains and NBRC 15308^T^, ANI and dDDH values were calculated for all combinations (Fig. S1). ANI ≥95% and dDDH ≥70% are classified as the same species (Goris *et al.*, 2007; Srivastava *et al.*, 2020). The ANI values for all combinations exceeded 95% identity, which is commonly used to define the same species, whereas those for combinations within the same AHL degradation group exceeded 97.5%. Furthermore, the dDDH values for combinations within the same AHL degradation group exceeded 75%. These results suggest that strains within the same AHL degradation group were related at the subspecies level. To compare the amino acid sequences of AiiB in the genome sequences from seven strains belonging to Groups 1 and 3, multiple sequence alignment was performed. Although there were slight substitutions of amino acid residues in AiiB between Group 1 and 3 strains, the overall amino acid sequences of AiiB were almost identical among the seven strains (Fig. S2). Therefore, the amino acid substitution and AHL-degrading activity of AiiB were assumed to be less relevant. *aiiB* and its surrounding sequences were then obtained from the genome sequences of *P. megaterium* strains, and differences between the three AHL degradation groups were compared. In the genome sequences of Group 1 and 3 strains, genes involved in the sugar diacid recognition domain-containing protein and sigma 54-interacting transcriptional regulator were placed upstream of *aiiB*, while the gene involved in iron-containing alcohol dehydrogenase was placed downstream of *aiiB* (Fig. 3). In contrast, although genes involved in the sugar diacid recognition domain-containing protein and iron-containing alcohol dehydrogenase were present in the genome sequence of Group 2 strains, *aiiB* and the sigma 54-interacting transcriptional regulator gene completely disappeared. Gene clusters upstream of *aiiB* were very similar among the three groups, whereas those downstream of *aiiB* were very similar between Groups 1 and 2, but markedly different for Group 3. We previously demonstrated that the extracellular AHL lactonase gene *qsdS* was placed between *leuC2* and *leuD* in the genome sequence of AHL-degrading *Sphingopixis* species, but was completely missing between *leuC2* and *leuD* in the genome sequences of *Sphingopyxis* species without AHL-degrading activity (Morohoshi *et al.*, 2019a). Since transposase-like sequences were not present around *aiiB*, it was assumed that *aiiB* and the sigma 54-interacting transcriptional regulator gene were obtained or missed by a non-transpositional event in the process of evolution in *P. megaterium*, similar to *Sphingopixis* species.

### Evaluation of AHL-degrading activity of the *aiiB* gene and *P. megaterium* strains

To investigate whether slight substitutions of amino acid residues in AiiB between Groups 1 and 3 affected AHL-degrading activity, the *aiiB*-coding region was amplified by PCR, cloned into the pUC118 vector, and transformed into *E. coli* DH5α. When the AHL-degrading activity of *E. coli* DH5α harboring *aiiB* from Groups 1 and 3 was evaluated, all strains completely degraded 20‍ ‍μM C6-HSL and C10-HSL within 1‍ ‍h (Fig. S3). These results demonstrated that AiiB from Group 3 exhibited high AHL-degrading activity, as did that from Group 1. Since Group 3 strains may exhibit AHL-degrading activity, the AHL-degrading activities of all selected strains of the three AHL degradation groups were exami­ned over a long incubation period. The results obtained are shown in Table 2. Similar to the results of the first screening, Group 1 strains completely degraded 20‍ ‍μL of C6-HSL within 3 h. In the case of Group 3 strains, only a slight reduction was observed in the concentration of C6-HSL after 3 h; however, it continued to decrease over the course of the incubation period and reached below the detection limit after 24 h. Since no significant difference was observed in the AHL-degrading activity of AiiB from Group 1 and 3 strains expressed in *E. coli* DH5α, the weak AHL-degrading activities of Group 3 strains may be due to the low expression level of AiiB rather than the amino acid substitution of AiiB. Future comparisons of *aiiB* expression levels among AHL degradation groups may reveal the reason for the weak AHL-degrading activities of Group 3 strains. Group 2 strains, which did not possess the *aiiB* gene, did not completely degrade 20‍ ‍μL of C6-HSL after 24 h; however, a slight decrease was noted in the concentration of C6-HSL. A previous study reported that cytochrome P450 from *P. megaterium* was capable of oxidizing AHLs and decreasing the strength of the quorum-sensing signal (Chowdhary *et al.*, 2007). In addition, AHLs are known to be unstable at high pH and are non-enzymatically hydrolyzed (Byers Joseph *et al.*, 2002). Therefore, the slight decrease observed in the concentration of C6-HSL in Group 2 strains may be attributed to pH changes and AHL oxidation rather than enzymatic hydrolysis by AHL lactonase.

### AiiB works as a quorum-quenching enzyme for the inhibition of plant pathogenic bacteria

**Fig. 4.**
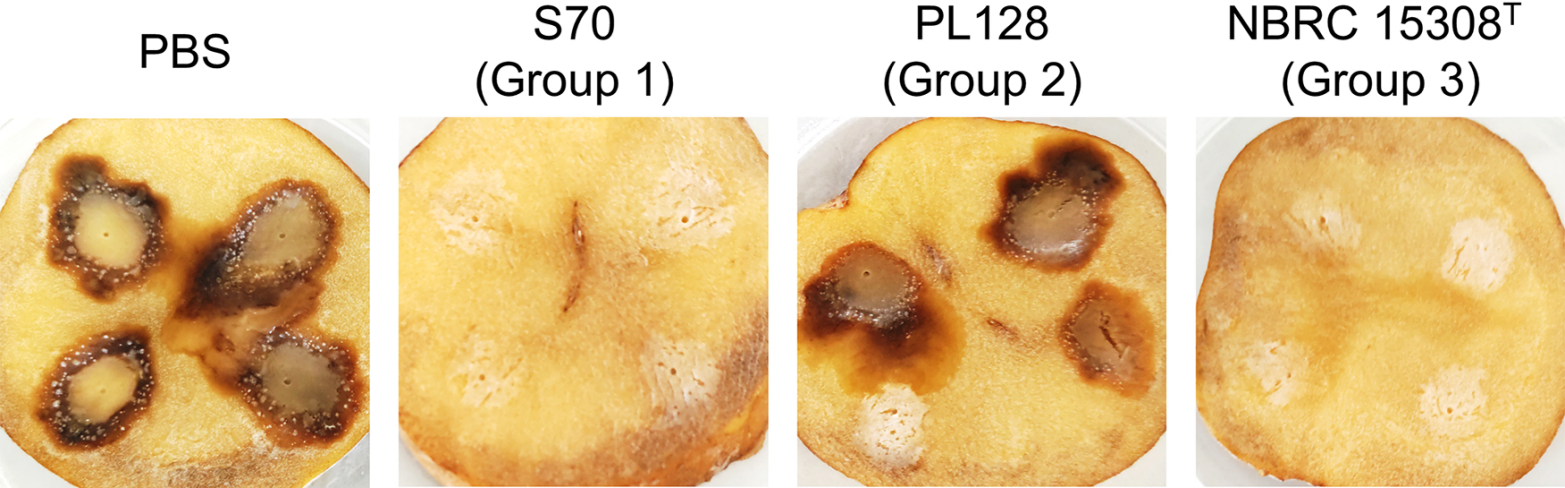
Attenuation of the maceration capacity of *P. carotovorum* subsp. *carotovorum* NBRC 12380 by *Priestia megaterium* strains. Full-grown cultures of the NBRC 12380 and *P. megaterium* strains were suspended in PBS. The cell suspension of NBRC 12380 was mixed with PBS or the cell suspension of S70, PL128, or NBRC 15308^T^. The mixture (10‍ ‍μL) was inoculated onto potato slices. After an incubation at 30°C for 2 days, the development of the maceration area was observed. This assay was repeated three times.

To assess the quorum-quenching ability of *P. megaterium*, *P. carotovorum* subsp. *carotovorum* NBRC 12380, which induces the production of various exoenzymes and plant tissue maceration by AHL-mediated quorum sensing, was used as a model plant pathogen (Morohoshi *et al.*, 2019b). The results of the potato slice maceration assay are shown in Fig. 4. When the cell suspension of NBRC 12380 was spotted onto potato slices, maceration symptoms of potato slices were clearly observed at inoculation sites after 2 days of incubation. Since PL128 (Group 2) did not exhibit AHL-degrading activity, potato slice maceration by NBRC 12380 was not attenuated by a co-inoculation with PL128. In contrast, a co-inoculation with S70 (Group 1) effectively attenuated maceration symptoms induced by NBRC 12380. Although the amelioration of maceration symptoms may be affected by nutrient competition, the AHL-degrading activity of S70 appeared to reduce the expression of exoenzymes controlled by quorum sensing in NBRC 12380 because only S70 with AHL-degrading activity attenuated maceration symptoms. NBRC 15308^T^ (Group 3), which exhibited weak AHL-degrading activity, also mitigated maceration symptoms induced by NBRC 12380. Therefore, the AHL-degrading activity of the Group 3 strain was very weak, but sufficient to inhibit the activation of quorum sensing in *P. carotovorum* subsp. *carotovorum*.

## Conclusion

We herein identified AHL-degrading activity and its distribution in *P. megaterium* strains and demonstrated its potential for the control of plant diseases based on the quorum-quenching strategy. A previous study demonstrated that cytochrome P450 from *P. megaterium* interfered with quorum sensing by oxidizing the acyl chain of AHL (Chowdhary *et al.*, 2007). However, oxidized AHLs were 18-fold less active than the parent compound, but retained quorum-sensing activity. In contrast, acylhomoserine, which is an AHL derivative that is hydrolyzed at its lactone ring, was not recognized as a quorum-sensing signaling compound. Therefore, the AHL lactonase activity of *P. megaterium* appears to contribute more strongly to the inhibition of quorum sensing in phytopathogenic bacteria than the AHL oxidation activity of cytochrome P450.

A previous study revealed that *P. megaterium* produced a number of secondary metabolites and exhibited biocontrol activity against plant pathogenic fungi, such as *Aspergillus* and *Fusarium* (Saleh *et al.*, 2021), and plant pathogenic nematodes, including *Meloidogyne graminicola* (Kildea *et al.*, 2008). In addition, *P. megaterium* RmBm31, an endophytic bacterium isolated from root nodules, was found to exhibit plant growth-promoting activity (Dahmani *et al.*, 2020). Although few studies have demonstrated the biocontrol activity of *P. megaterium* against plant pathogenic bacteria, the AHL-degrading activity of *P. megaterium* may effectively inhibit the AHL-mediated expression of virulence factors in phytopathogenic bacteria. If the selection of AHL-degrading *P. megaterium* strains that produce anti­nematodal and antifungal secondary metabolites and exhibit plant growth-promoting activities is possible, they have potential as multifunctional biocontrol agents that may also control bacterial diseases.

## Citation

Morohoshi, T., Arai, W., Ueno, K., and Someya, N. (2025) Distribution and Characterization of the Novel Quorum-quenching Enzyme AiiB in *Priestia megaterium* Isolated from a Natural Environment. *Microbes Environ ***40**: ME25004.

https://doi.org/10.1264/jsme2.ME25004

## Supplementary Material

Supplementary Material

## Figures and Tables

**Table 1.** AHL degradation and presence of the *aiiB* gene in *Priestia megaterium* strains

Group	AHL degradation^a^	*aiiB* gene	Strain numbers		
			Soil	Leaf	Root
1	+	+	58	20	19
2	–	–	263	135	159
3	–	+	25	17	26
Total			346	172	204

^a^ The plus sign means the complete degradation of 20‍ ‍μM C6-HSL after a 3-h incubation

**Table 2.** Time course for the degradation of C6-HSL by *Priestia megaterium* strains

Strains	AHL degradation group	C6-HSL concentration (nM)^a^			
		3 h	6 h	9 h	24 h
S70	1	BD	—	—	—
PL125	1	BD	—	—	—
PR185	1	BD	—	—	—
S67	2	ND	ND	ND	7.0±2.6
PL128	2	ND	ND	ND	7.0±1.2
PR54	2	ND	ND	ND	5.1±0.4
S228	3	19.4±0.2	11.5±0.4	6.3±0.3	BD
PL103	3	11.6±0.7	7.7±0.6	3.4±0.6	BD
PR236	3	9.4±0.2	6.2±0.3	3.6±0.4	BD
NBRC 15308^T^	3	9.9±0.6	6.3±0.5	4.4±0.6	BD

^a^ BD, below the detection limit. ND, not determined.
