# Supplementary material for: Distribution and Characterization of the Novel Quorum-quenching Enzyme AiiB in Priestia megaterium Isolated from a Natural Environment
Source: Microbes Environ. 2025 Jul 18;40(3):ME25004. doi: 10.1264/jsme2.ME25004 (PMC12501872; doi:10.1264/jsme2.ME25004)
Supplement: Supplementary file 1 — Supplementary Material [file 40_25004_s1.pdf]

Strains		Group 1			Group 2			Group 3			
		S70	PL125	PR185	S67	PL128	PR54	S228	PL103	PR236	NBRC 15308^T
Group 1	S70	–	98.4	98.4	95.3	95.5	95.4	95.5	95.4	95.4	95.5
	PL125	85.8	–	98.5	95.6	95.6	95.5	95.6	95.4	95.5	95.6
	PR185	86.0	87.4	–	95.5	95.5	95.4	95.5	95.5	95.5	95.5
Group 2	S67	63.1	64.2	63.5	–	99.5	99.6	96.9	96.7	97.0	96.8
	PL128	63.6	64.3	63.4	95.1	–	99.5	96.8	96.7	97.0	96.9
	PR54	63.3	64.0	63.5	96.2	95.5	–	96.8	96.7	97.1	96.9
Group 3	S228	63.6	64.1	63.7	72.6	72.9	72.5	–	99.2	97.7	99.2
	PL103	63.4	63.9	63.3	71.0	71.1	71.2	93.1	–	97.7	99.5
	PR236	63.5	64.3	63.9	74.2	74.1	74.4	79.4	78.7	–	97.8
	NBRC 15308^T	64.2	64.5	64.3	73.3	73.7	73.6	93.4	96.2	80.2	–

ANI	>99%	>97.5%	>95%

dDDH	>90%	>75%	>70%	>60%

Fig. S1. Calculation results of the average nucleotide identity (ANI) (Yoon *et al.*, 2017) and digital DNA-DNA hybridization (dDDH) (Meier-Kolthoff *et al.*, 2022) values for all combinations of selected *Priestia megaterium* strains.

References

Meier-Kolthoff, J.P., Carbasse, J.S., Peinado-Olarte, R.L., and Göker, M. (2022) TYGS and LPSN: a database tandem for fast and reliable genome-based classification and nomenclature of prokaryotes. Nucleic Acids Res 50: D801-D807.

Yoon, S.H., Ha, S.M., Lim, J., Kwon, S., and Chun, J. (2017) A large-scale evaluation of algorithms to calculate average nucleotide identity. Antonie Van Leeuwenhoek 110: 1281-1286.

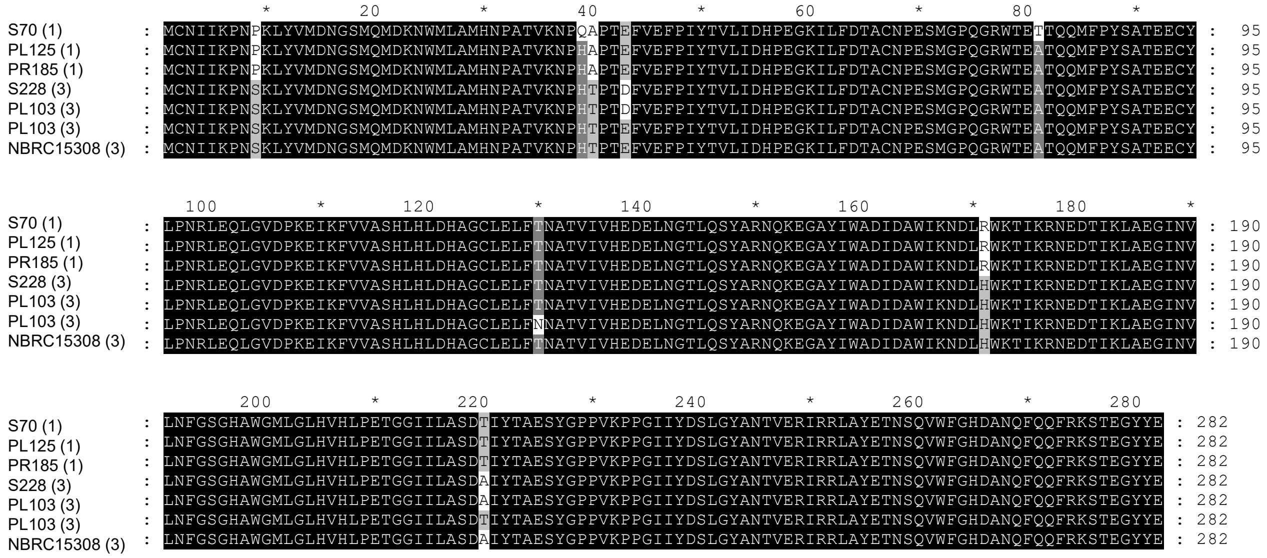

Fig. S2. Comparison of amino acid sequences of AiiB from *Priestia megaterium* strains. The AHL-degrading group numbers are listed in parentheses. Sequences were aligned using ClustalW (Thompson *et al.* 1994) and shaded using GeneDoc software (https://nrbsc.org/gfx/genedoc).

References

Thompson, J.D., D.G. Higgins, and T.J. Gibson. 1994. CLUSTAL W: improving the sensitivity of progressive multiple sequence alignment through sequence weighting, position-specific gap penalties and weight matrix choice. Nucleic Acids Res. 22:4673-4680.

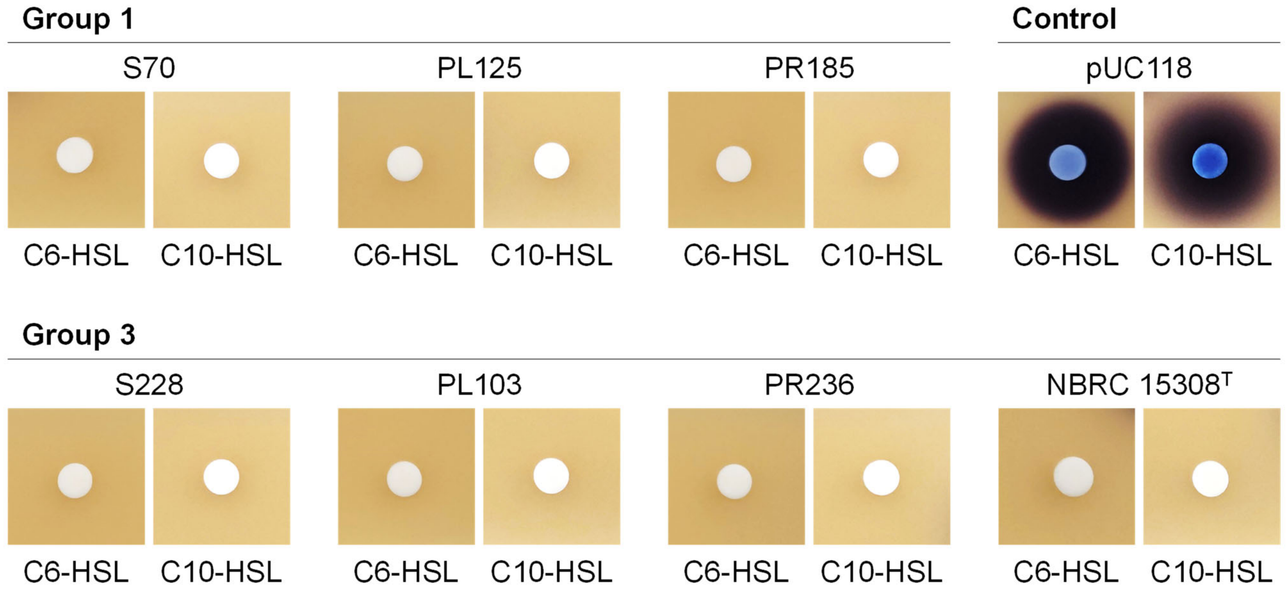

Fig. S3. AHL-degrading activity of *Escherichia coli* DH5α harboring *aiiB* from *Priestia megatherium* Group 1 and 3 strains. *E. coli* DH5α harboring pUC118 vector was used for control without AHL-degrading activity. Full-grown cultures of *E. coli* DH5α harboring *aiiB* were mixed with C6-HSL or C10-HSL at a final concentration of 20 μM. After incubation at 30°C for 1 h with shaking, the remaining AHL was detected as a purple pigment on LB agar medium containing *Chromobacterium violaceum* CV026 for C6-HSL (McClean *et al.* 1997) or VIR07 for C10-HSL (Morohoshi *et al.* 2008).

References

McClean, K.H., Winson, M.K., Fish, L., Taylor, A., Chhabra, S.R., Camara, M. et al. (1997) Quorum sensing and *Chromobacterium violaceum*: exploitation of violacein production and inhibition for the detection of *N*-acylhomoserine lactones. Microbiology 143: 3703–3711.

Morohoshi, T., Kato, M., Fukamachi, K., Kato, N., and Ikeda, T. (2008) *N*-acylhomoserine lactone regulates violacein production in *Chromobacterium violaceum* type strain ATCC 12472. FEMS Microbiol Lett 279: 124–130.

Table S1. Primers used in this study

Description	Primers	Primer sequences (5'-3')*
Amplification of internal region of *aiiB*	AiiBp-F	GCATAACCCAGCAACTGTTAAGAACCC
	AiiBp-R	GCATCGTGACCAAACCAAACTTGCG
Amplification of *aiiB* for cloning	Pme_aiiB-F1	TCTGAATTCAAGCGGTGAATACTTTCAGCACATGG
	Pme_aiiB-R1	TCTCTGCAGGAAGCGATCCCCATCCTGTATAGCTG
Amplification of *aiiB* for cloning (PL125 and PR185)	Pme_aiiB-F2	TCTGAATTCTCCGCGCTTGGTTATAAGAACCAAGG
	Pme_aiiB-R2	TCTGGATCCTGATCCCCATCCTGTGTAGCTAAGAG

*Restriction sites are underlined.

Table S2. Source and AHL-degrading properties of *Priestia megaterium* strains used in this study

Strains	AHL degradation Group	C6-HSL degradation (3 h)	*aiiB* gene	Source	Site / Plant
NBRC 15308^{T}	3	–	+	Soil	–
S6	2	–	–	Soil	Ibaraki
S8	2	–	–	Soil	Ibaraki
S9	2	–	–	Soil	Ibaraki
S12	2	–	–	Soil	Tokyo
S13	2	–	–	Soil	Tokyo
S15	2	–	–	Soil	Tokyo
S19	2	–	–	Soil	Tokyo
S20	2	–	–	Soil	Tokyo
S21	2	–	–	Soil	Tokyo
S22	2	–	–	Soil	Tokyo
S26	2	–	–	Soil	Tokyo
S29	2	–	–	Soil	Tokyo
S32	2	–	–	Soil	Ibaraki
S34	2	–	–	Soil	Ibaraki
S35	2	–	–	Soil	Ibaraki
S37	3	–	+	Soil	Ibaraki
S39	2	–	–	Soil	Ibaraki
S41	2	–	–	Soil	Ibaraki
S42	3	–	+	Soil	Ibaraki
S43	2	–	–	Soil	Ibaraki
S44	2	–	–	Soil	Ibaraki
S45	1	+	+	Soil	Ibaraki
S47	2	–	–	Soil	Ibaraki
S54	2	–	–	Soil	Ibaraki
S58	2	–	–	Soil	Ibaraki
S60	1	+	+	Soil	Ibaraki
S63	2	–	–	Soil	Ibaraki
S65	2	–	–	Soil	Ibaraki
S67	2	–	–	Soil	Ibaraki
S69	2	–	–	Soil	Ibaraki
S70	1	+	+	Soil	Ibaraki
S73	3	–	+	Soil	Ibaraki
S74	2	–	–	Soil	Ibaraki
S78	2	–	–	Soil	Ibaraki
S85	2	–	–	Soil	Ibaraki
S87	2	–	–	Soil	Ibaraki
S90	2	–	–	Soil	Ibaraki

S97	2	–	–	Soil	Ibaraki
S99	2	–	–	Soil	Ibaraki
S100	2	–	–	Soil	Ibaraki
S102	2	–	–	Soil	Ibaraki
S103	2	–	–	Soil	Ibaraki
S104	2	–	–	Soil	Ibaraki
S107	2	–	–	Soil	Ibaraki
S108	2	–	–	Soil	Ibaraki
S115	2	–	–	Soil	Ibaraki
S117	2	–	–	Soil	Ibaraki
S119	2	–	–	Soil	Ibaraki
S120	2	–	–	Soil	Ibaraki
S125	2	–	–	Soil	Ibaraki
S127	2	–	–	Soil	Ibaraki
S131	2	–	–	Soil	Ibaraki
S140	1	+	+	Soil	Ibaraki
S145	2	–	–	Soil	Ibaraki
S151	2	–	–	Soil	Ibaraki
S156	2	–	–	Soil	Ibaraki
S157	2	–	–	Soil	Ibaraki
S161	2	–	–	Soil	Ibaraki
S165	3	–	+	Soil	Ibaraki
S167	2	–	–	Soil	Ibaraki
S170	2	–	–	Soil	Ibaraki
S176	2	–	–	Soil	Ibaraki
S177	2	–	–	Soil	Ibaraki
S179	2	–	–	Soil	Ibaraki
S191	2	–	–	Soil	Ibaraki
S192	2	–	–	Soil	Ibaraki
S196	2	–	–	Soil	Ibaraki
S198	1	+	+	Soil	Ibaraki
S203	1	+	+	Soil	Ibaraki
S205	2	–	–	Soil	Ibaraki
S206	2	–	–	Soil	Ibaraki
S208	2	–	–	Soil	Ibaraki
S210	1	+	+	Soil	Ibaraki
S211	2	–	–	Soil	Tochigi
S214	2	–	–	Soil	Tochigi
S217	2	–	–	Soil	Tochigi
S219	2	–	–	Soil	Tochigi
S223	2	–	–	Soil	Tochigi
S227	2	–	–	Soil	Tochigi

S228	3	–	+	Soil	Tochigi
S241	2	–	–	Soil	Tochigi
S248	2	–	–	Soil	Tochigi
S259	2	–	–	Soil	Tochigi
S265	1	+	+	Soil	Tochigi
S266	2	–	–	Soil	Tochigi
S267	1	+	+	Soil	Tochigi
S269	2	−	–	Soil	Tochigi
S272	1	+	+	Soil	Tochigi
S273	2	–	–	Soil	Tochigi
S278	2	–	–	Soil	Tochigi
S281	3	–	+	Soil	Tochigi
S282	2	–	–	Soil	Tochigi
S284	3	–	+	Soil	Tochigi
S289	2	–	–	Soil	Tochigi
S292	2	–	–	Soil	Ibaraki
S293	2	–	–	Soil	Ibaraki
S299	2	–	–	Soil	Ibaraki
S301	1	+	+	Soil	Ibaraki
S302	2	–	–	Soil	Ibaraki
S305	2	–	–	Soil	Ibaraki
S306	1	+	+	Soil	Ibaraki
S307	2	–	–	Soil	Ibaraki
S308	2	–	–	Soil	Ibaraki
S311	1	+	+	Soil	Ibaraki
S313	2	–	–	Soil	Ibaraki
S315	1	+	+	Soil	Ibaraki
S316	2	–	–	Soil	Ibaraki
S317	2	–	–	Soil	Ibaraki
S319	2	–	–	Soil	Ibaraki
S324	2	–	–	Soil	Ibaraki
S325	1	+	+	Soil	Ibaraki
S328	1	+	+	Soil	Ibaraki
S329	2	–	–	Soil	Ibaraki
S330	2	–	–	Soil	Ibaraki
S331	1	+	+	Soil	Ibaraki
S332	1	+	+	Soil	Ibaraki
S333	2	–	–	Soil	Ibaraki
S335	2	–	–	Soil	Ibaraki
S336	2	–	–	Soil	Ibaraki
S341	2	–	–	Soil	Ibaraki
S343	2	–	–	Soil	Ibaraki

S344	2	–	–	Soil	Ibaraki
S349	1	+	+	Soil	Ibaraki
S354	3	–	+	Soil	Ibaraki
S355	1	+	+	Soil	Ibaraki
S356	2	–	–	Soil	Ibaraki
S357	2	–	–	Soil	Ibaraki
S359	2	–	–	Soil	Ibaraki
S360	1	+	+	Soil	Ibaraki
S362	2	–	–	Soil	Ibaraki
S363	2	–	–	Soil	Ibaraki
S364	2	–	–	Soil	Ibaraki
S365	2	–	–	Soil	Ibaraki
S370	2	–	–	Soil	Ibaraki
S374	3	–	+	Soil	Ibaraki
S376	2	–	–	Soil	Ibaraki
S377	2	–	–	Soil	Ibaraki
S380	2	–	–	Soil	Ibaraki
S384	3	–	+	Soil	Tokyo
S385	2	–	–	Soil	Tokyo
S388	2	–	–	Soil	Tokyo
S394	2	–	–	Soil	Tokyo
S395	3	–	+	Soil	Tokyo
S396	3	–	+	Soil	Tokyo
S397	2	–	–	Soil	Tokyo
S398	3	–	+	Soil	Tokyo
S399	3	–	+	Soil	Tokyo
S406	1	+	+	Soil	Tokyo
S407	2	–	–	Soil	Tokyo
S408	3	–	+	Soil	Tokyo
S409	2	–	–	Soil	Tokyo
S412	2	–	–	Soil	Tokyo
S416	1	+	+	Soil	Tokyo
S418	1	+	+	Soil	Tokyo
S419	2	–	–	Soil	Tokyo
S421	2	–	–	Soil	Ibaraki
S422	2	–	–	Soil	Ibaraki
S424	2	–	–	Soil	Ibaraki
S426	2	–	–	Soil	Ibaraki
S435	2	–	–	Soil	Ibaraki
S436	2	–	–	Soil	Ibaraki
S438	2	–	–	Soil	Ibaraki
S443	2	–	–	Soil	Ibaraki

S445	1	+	+	Soil	Ibaraki
S446	2	–	–	Soil	Ibaraki
S449	3	–	+	Soil	Ibaraki
S450	2	–	–	Soil	Ibaraki
S456	2	–	–	Soil	Ibaraki
S457	2	–	–	Soil	Ibaraki
S458	1	+	+	Soil	Ibaraki
S459	2	–	–	Soil	Ibaraki
S460	2	–	–	Soil	Ibaraki
S466	2	–	–	Soil	Ibaraki
S468	2	–	–	Soil	Ibaraki
S469	2	–	–	Soil	Ibaraki
S473	2	–	–	Soil	Tokyo
S475	2	–	–	Soil	Tokyo
S476	2	–	–	Soil	Tokyo
S478	2	–	–	Soil	Tokyo
S479	2	–	–	Soil	Tokyo
S480	2	–	–	Soil	Tokyo
S483	2	−	–	Soil	Tokyo
S485	2	–	–	Soil	Tokyo
S486	1	+	+	Soil	Tokyo
S487	2	–	–	Soil	Tokyo
S488	2	–	–	Soil	Tokyo
S489	2	–	–	Soil	Tokyo
S490	2	–	–	Soil	Tokyo
S495	1	+	+	Soil	Tokyo
S496	1	+	+	Soil	Tokyo
S498	2	–	–	Soil	Tokyo
S499	2	–	–	Soil	Tokyo
S500	2	–	–	Soil	Tokyo
S506	2	–	–	Soil	Ibaraki
S507	2	–	–	Soil	Ibaraki
S509	2	–	–	Soil	Ibaraki
S512	2	–	–	Soil	Tochigi
S516	1	+	+	Soil	Tochigi
S518	2	–	–	Soil	Tochigi
S530	2	–	–	Soil	Tochigi
S538	2	–	–	Soil	Tochigi
S539	2	–	–	Soil	Tochigi
S542	2	–	–	Soil	Tochigi
S545	2	–	–	Soil	Tochigi
S546	2	–	–	Soil	Tochigi

S547	1	+	+	Soil	Tochigi
S548	1	+	+	Soil	Tochigi
S554	1	+	+	Soil	Tochigi
S555	1	+	+	Soil	Tochigi
S556	2	–	–	Soil	Tochigi
S557	2	–	–	Soil	Tochigi
S565	2	–	–	Soil	Tochigi
S566	1	+	+	Soil	Tochigi
S567	2	–	–	Soil	Tochigi
S574	2	–	–	Soil	Ibaraki
S576	2	–	–	Soil	Ibaraki
S586	2	–	–	Soil	Ibaraki
S594	2	–	–	Soil	Ibaraki
S595	2	–	–	Soil	Ibaraki
S596	1	+	+	Soil	Ibaraki
S597	2	–	–	Soil	Ibaraki
S605	2	–	–	Soil	Ibaraki
S606	2	–	–	Soil	Ibaraki
S614	2	–	–	Soil	Ibaraki
S625	2	–	–	Soil	Tokyo
S626	2	–	–	Soil	Tokyo
S627	1	+	+	Soil	Tokyo
S628	2	–	–	Soil	Tokyo
S629	3	–	+	Soil	Tokyo
S636	2	–	–	Soil	Ibaraki
S637	1	+	+	Soil	Ibaraki
S644	2	–	–	Soil	Ibaraki
S645	2	–	–	Soil	Ibaraki
S646	2	–	–	Soil	Ibaraki
S654	2	–	–	Soil	Fukushima
S655	2	–	–	Soil	Fukushima
S656	2	–	–	Soil	Fukushima
S665	2	–	–	Soil	Fukushima
S666	1	+	+	Soil	Fukushima
S667	2	–	–	Soil	Fukushima
S675	1	+	+	Soil	Fukushima
S676	2	–	–	Soil	Fukushima
S683	2	–	–	Soil	Fukushima
S693	1	+	+	Soil	Fukushima
S694	2	–	–	Soil	Fukushima
S695	2	–	–	Soil	Fukushima
S705	2	–	–	Soil	Fukushima

S712	3	–	+	Soil	Ibaraki
S725	2	–	–	Soil	Ibaraki
S728	2	–	–	Soil	Ibaraki
S736	1	+	+	Soil	Ibaraki
S737	1	+	+	Soil	Ibaraki
S738	2	–	–	Soil	Ibaraki
S747	1	+	+	Soil	Ibaraki
S766	1	+	+	Soil	Ibaraki
S767	3	–	+	Soil	Ibaraki
S772	2	–	–	Soil	Fukushima
S773	2	–	–	Soil	Fukushima
S776	1	+	+	Soil	Fukushima
S786	2	–	–	Soil	Ibaraki
S787	2	–	–	Soil	Ibaraki
S796	2	–	–	Soil	Ibaraki
S806	1	+	+	Soil	Ibaraki
S807	2	–	–	Soil	Ibaraki
S808	2	–	–	Soil	Ibaraki
S815	2	–	–	Soil	Tokyo
S816	2	–	–	Soil	Tokyo
S825	2	–	–	Soil	Tokyo
S833	2	–	–	Soil	Tokyo
S834	1	+	+	Soil	Tokyo
S845	2	–	–	Soil	Tokyo
S846	2	–	–	Soil	Tokyo
S854	2	–	–	Soil	Tokyo
S856	1	+	+	Soil	Tokyo
S857	2	–	–	Soil	Tokyo
S865	2	–	–	Soil	Tokyo
S866	2	–	–	Soil	Tokyo
S873	2	–	–	Soil	Tokyo
S875	1	+	+	Soil	Tokyo
S876	2	–	–	Soil	Tokyo
S877	2	–	–	Soil	Tokyo
S886	2	–	–	Soil	Tokyo
S894	2	–	–	Soil	Ibaraki
S896	3	–	+	Soil	Ibaraki
S906	2	–	–	Soil	Chiba
S916	2	–	–	Soil	Chiba
S917	2	–	–	Soil	Chiba
S924	2	–	–	Soil	Chiba
S925	2	–	–	Soil	Chiba

S926	2	–	–	Soil	Chiba
S935	2	–	–	Soil	Chiba
S937	2	–	–	Soil	Chiba
S948	2	–	–	Soil	Chiba
S956	2	–	–	Soil	Chiba
S958	2	–	–	Soil	Chiba
S965	2	–	–	Soil	Chiba
S966	2	–	–	Soil	Chiba
S967	2	–	–	Soil	Chiba
S975	2	–	–	Soil	Chiba
S976	1	+	+	Soil	Chiba
S977	2	–	–	Soil	Chiba
S985	2	–	–	Soil	Chiba
S986	3	–	+	Soil	Chiba
S988	2	–	–	Soil	Chiba
S1007	2	–	–	Soil	Kagoshima
S1016	2	–	–	Soil	Kagoshima
S1025	2	–	–	Soil	Kagoshima
S1027	3	–	+	Soil	Kagoshima
S1035	2	–	–	Soil	Kagoshima
S1044	2	–	–	Soil	Kagoshima
S1056	2	–	–	Soil	Kagoshima
S1064	3	–	+	Soil	Kagoshima
S1073	1	+	+	Soil	Kagoshima
S1074	2	–	–	Soil	Kagoshima
S1086	1	+	+	Soil	Kagoshima
S1108	2	–	–	Soil	Kagoshima
S1114	2	–	–	Soil	Kagoshima
S1116	1	+	+	Soil	Kagoshima
S1123	2	–	–	Soil	Kagoshima
S1134	2	–	–	Soil	Kagoshima
S1135	2	–	–	Soil	Kagoshima
S1146	2	–	–	Soil	Kagoshima
S1156	2	–	–	Soil	Kagoshima
S1165	2	–	–	Soil	Kagoshima
S1166	1	+	+	Soil	Kagoshima
S1185	2	–	–	Soil	Kagoshima
S1186	2	–	–	Soil	Kagoshima
S1193	1	+	+	Soil	Chiba
S1194	2	–	–	Soil	Chiba
S1205	3	–	+	Soil	Chiba
S1206	2	–	–	Soil	Chiba

S1216	2	–	–	Soil	Chiba
S1233	2	–	–	Soil	Ibaraki
S1235	1	+	+	Soil	Ibaraki
S1245	2	–	–	Soil	Ibaraki
S1246	2	–	–	Soil	Ibaraki
S1253	2	–	–	Soil	Kumamoto
S1254	2	–	–	Soil	Kumamoto
S1255	3	–	+	Soil	Kumamoto
S1263	2	–	–	Soil	Tokyo
S1266	1	+	+	Soil	Tokyo
S1267	2	–	–	Soil	Tokyo
S1284	2	–	–	Soil	Kanagawa
S1285	2	–	–	Soil	Kanagawa
S1297	2	–	–	Soil	Kanagawa
S1298	2	–	–	Soil	Kanagawa
PL3	2	–	–	Leaf	Leek
PL7	1	+	+	Leaf	Leek
PL9	1	+	+	Leaf	Leek
PL14	2	–	–	Leaf	Cabbage
PL15	2	–	–	Leaf	Cabbage
PL18	2	–	–	Leaf	Cabbage
PL19	2	–	–	Leaf	Cabbage
PL21	2	–	–	Leaf	Leek
PL22	2	–	–	Leaf	Leek
PL23	2	–	–	Leaf	Leek
PL24	2	–	–	Leaf	Leek
PL27	2	–	–	Leaf	Leek
PL29	2	–	–	Leaf	Leek
PL33	1	+	+	Leaf	Japanese radish
PL34	2	–	–	Leaf	Japanese radish
PL37	2	–	–	Leaf	Japanese radish
PL38	2	–	–	Leaf	Japanese radish
PL39	2	–	–	Leaf	Japanese radish
PL44	2	–	–	Leaf	Spinach
PL45	2	–	–	Leaf	Spinach
PL47	2	–	–	Leaf	Spinach
PL50	2	–	–	Leaf	Spinach
PL53	2	–	–	Leaf	Spinach
PL55	2	–	–	Leaf	Spinach
PL56	2	–	–	Leaf	Spinach
PL57	2	–	–	Leaf	Spinach
PL59	2	–	–	Leaf	Spinach

PL63	2	–	–	Leaf	Spinach
PL64	2	–	–	Leaf	Spinach
PL66	2	–	–	Leaf	Spinach
PL67	2	–	–	Leaf	Spinach
PL68	2	–	–	Leaf	Spinach
PL70	2	–	–	Leaf	Spinach
PL74	3	–	+	Leaf	Spinach
PL75	2	–	–	Leaf	Spinach
PL76	3	–	+	Leaf	Spinach
PL77	1	+	+	Leaf	Spinach
PL82	2	–	–	Leaf	Spinach
PL84	1	+	+	Leaf	Spinach
PL85	2	–	–	Leaf	Spinach
PL86	2	–	–	Leaf	Spinach
PL92	2	–	–	Leaf	Spinach
PL102	2	–	–	Leaf	Spinach
PL103	3	–	+	Leaf	Spinach
PL107	3	–	+	Leaf	Spinach
PL108	2	–	–	Leaf	Spinach
PL109	1	+	+	Leaf	Spinach
PL110	2	–	–	Leaf	Spinach
PL113	2	–	–	Leaf	Spinach
PL116	2	–	–	Leaf	Spinach
PL118	2	–	–	Leaf	Spinach
PL124	2	–	–	Leaf	Spinach
PL125	1	+	+	Leaf	Spinach
PL126	2	–	–	Leaf	Spinach
PL127	2	–	–	Leaf	Spinach
PL128	2	–	–	Leaf	Spinach
PL129	2	–	–	Leaf	Spinach
PL132	2	–	–	Leaf	Cabbage
PL136	2	–	–	Leaf	Cabbage
PL141	2	–	–	Leaf	Chinese cabbage
PL146	2	–	–	Leaf	Chinese cabbage
PL147	2	–	–	Leaf	Chinese cabbage
PL148	2	–	–	Leaf	Chinese cabbage
PL149	1	+	+	Leaf	Chinese cabbage
PL152	2	–	–	Leaf	Chinese chives
PL154	2	–	–	Leaf	Chinese chives
PL156	3	–	+	Leaf	Chinese chives
PL158	2	–	–	Leaf	Chinese chives
PL164	1	+	+	Leaf	Crown daisy

PL165	2	–	–	Leaf	Crown daisy
PL166	2	–	–	Leaf	Crown daisy
PL167	2	–	–	Leaf	Crown daisy
PL168	2	–	–	Leaf	Crown daisy
PL175	2	–	–	Leaf	Broccoli
PL176	2	–	–	Leaf	Broccoli
PL177	2	–	–	Leaf	Broccoli
PL178	2	–	–	Leaf	Broccoli
PL184	2	–	–	Leaf	Spinach
PL185	2	–	–	Leaf	Spinach
PL186	2	–	–	Leaf	Spinach
PL187	2	–	–	Leaf	Spinach
PL188	2	–	–	Leaf	Spinach
PL189	2	–	–	Leaf	Spinach
PL192	2	–	–	Leaf	Spinach
PL193	2	–	–	Leaf	Spinach
PL194	2	–	–	Leaf	Spinach
PL197	3	–	+	Leaf	Spinach
PL198	2	–	–	Leaf	Spinach
PL204	2	–	–	Leaf	Spinach
PL205	1	+	+	Leaf	Spinach
PL206	2	–	–	Leaf	Spinach
PL207	2	–	–	Leaf	Spinach
PL208	2	–	–	Leaf	Spinach
PL214	2	–	–	Leaf	Spinach
PL215	2	–	–	Leaf	Spinach
PL216	3	–	+	Leaf	Spinach
PL217	2	–	–	Leaf	Spinach
PL222	2	–	–	Leaf	Chinese chives
PL223	2	–	–	Leaf	Chinese chives
PL226	2	–	–	Leaf	Chinese chives
PL227	2	–	–	Leaf	Chinese chives
PL233	2	–	–	Leaf	Chinese cabbage
PL235	2	–	–	Leaf	Chinese cabbage
PL236	2	–	–	Leaf	Chinese cabbage
PL237	1	+	+	Leaf	Chinese cabbage
PL242	3	–	+	Leaf	Leek
PL245	2	–	–	Leaf	Leek
PL246	2	–	–	Leaf	Leek
PL247	2	–	–	Leaf	Leek
PL248	2	–	–	Leaf	Leek
PL254	3	–	+	Leaf	Spinach

PL257	1	+	+	Leaf	Spinach
PL258	1	+	+	Leaf	Spinach
PL259	2	–	–	Leaf	Spinach
PL262	2	–	–	Leaf	Leek
PL263	2	–	–	Leaf	Leek
PL266	2	–	–	Leaf	Leek
PL267	1	+	+	Leaf	Leek
PL268	2	–	–	Leaf	Leek
PL269	2	–	–	Leaf	Leek
PL270	2	–	–	Leaf	Leek
PL274	3	–	+	Leaf	Cabbage
PL276	1	+	+	Leaf	Cabbage
PL286	3	–	+	Leaf	Spinach
PL287	1	+	+	Leaf	Spinach
PL289	1	+	+	Leaf	Spinach
PL301	2	–	–	Leaf	Leek
PL305	2	–	–	Leaf	Leek
PL308	2	–	–	Leaf	Leek
PL315	3	–	+	Leaf	Japanese mustard spinach
PL316	2	–	–	Leaf	Japanese mustard spinach
PL326	2	–	–	Leaf	Carrot
PL327	1	+	+	Leaf	Carrot
PL328	2	–	–	Leaf	Carrot
PL334	2	–	–	Leaf	Lettuce
PL336	3	–	+	Leaf	Lettuce
PL343	2	–	–	Leaf	Broccoli
PL354	2	–	–	Leaf	Spinach
PL355	3	–	+	Leaf	Spinach
PL356	2	–	–	Leaf	Spinach
PL358	2	–	–	Leaf	Spinach
PL364	1	+	+	Leaf	Broccoli
PL366	2	–	–	Leaf	Broccoli
PL376	2	–	–	Leaf	Japanese radish
PL384	3	–	+	Leaf	Spinach
PL385	2	–	–	Leaf	Spinach
PL394	2	–	–	Leaf	Spinach
PL401	2	–	–	Leaf	Chinese chives
PL402	2	–	–	Leaf	Chinese chives
PL403	2	–	–	Leaf	Chinese chives
PL404	2	–	–	Leaf	Chinese chives
PL405	2	–	–	Leaf	Chinese chives
PL406	2	–	–	Leaf	Chinese chives

PL415	2	–	–	Leaf	Turnip
PL427	2	–	–	Leaf	Japanese mustard spinach
PL428	1	+	+	Leaf	Japanese mustard spinach
PL444	2	–	–	Leaf	Crown daisy
PL445	2	–	–	Leaf	Crown daisy
PL446	3	–	+	Leaf	Crown daisy
PL447	2	–	–	Leaf	Crown daisy
PL456	3	–	+	Leaf	Chinese cabbage
PL473	2	–	–	Leaf	Japanese radish
PL474	2	–	–	Leaf	Japanese radish
PL475	2	–	–	Leaf	Japanese radish
PL476	2	–	–	Leaf	Japanese radish
PL485	2	–	–	Leaf	Chinese cabbage
PL486	2	–	–	Leaf	Chinese cabbage
PL487	2	–	–	Leaf	Chinese cabbage
PL493	2	–	–	Leaf	Crown daisy
PL494	2	–	–	Leaf	Crown daisy
PL495	2	–	–	Leaf	Crown daisy
PL496	2	–	–	Leaf	Crown daisy
PR1	2	–	–	Root	Cabbage
PR2	2	–	–	Root	Cabbage
PR6	2	–	–	Root	Cabbage
PR8	2	–	–	Root	Cabbage
PR9	2	–	–	Root	Cabbage
PR11	2	–	–	Root	Spinach
PR12	2	–	–	Root	Spinach
PR14	2	–	–	Root	Spinach
PR15	2	–	–	Root	Spinach
PR16	2	–	–	Root	Spinach
PR25	2	–	–	Root	Spinach
PR31	2	–	–	Root	Spinach
PR32	2	–	–	Root	Spinach
PR34	2	–	–	Root	Spinach
PR36	2	–	–	Root	Spinach
PR42	2	–	–	Root	Spinach
PR43	2	–	–	Root	Spinach
PR47	2	–	–	Root	Spinach
PR51	2	–	–	Root	Spinach
PR52	2	–	–	Root	Spinach
PR54	2	–	–	Root	Spinach
PR57	2	–	–	Root	Spinach
PR72	2	–	–	Root	Spinach

PR81	2	–	–	Root	Spinach
PR82	2	–	–	Root	Spinach
PR87	2	–	–	Root	Spinach
PR88	2	–	–	Root	Spinach
PR90	2	–	–	Root	Spinach
PR91	2	–	–	Root	Leek
PR92	2	–	–	Root	Leek
PR95	2	–	–	Root	Leek
PR98	2	–	–	Root	Leek
PR103	1	+	+	Root	Spinach
PR106	1	+	+	Root	Spinach
PR107	2	–	–	Root	Spinach
PR109	2	–	–	Root	Spinach
PR110	2	–	–	Root	Spinach
PR114	1	+	+	Root	Spinach
PR117	2	–	–	Root	Spinach
PR118	2	–	–	Root	Spinach
PR120	2	–	–	Root	Spinach
PR121	2	–	–	Root	Spinach
PR122	2	–	–	Root	Spinach
PR124	2	–	–	Root	Spinach
PR128	1	+	+	Root	Spinach
PR130	2	–	–	Root	Spinach
PR134	2	–	–	Root	Spinach
PR143	2	–	–	Root	Spinach
PR145	1	+	+	Root	Spinach
PR146	2	–	–	Root	Spinach
PR147	2	–	–	Root	Spinach
PR150	1	+	+	Root	Spinach
PR152	3	–	+	Root	Spinach
PR156	1	+	+	Root	Spinach
PR157	2	–	–	Root	Spinach
PR158	2	–	–	Root	Spinach
PR159	2	–	–	Root	Spinach
PR160	2	–	–	Root	Spinach
PR161	2	–	–	Root	Sweet potato
PR162	2	–	–	Root	Sweet potato
PR163	2	–	–	Root	Sweet potato
PR165	2	–	–	Root	Sweet potato
PR166	2	–	–	Root	Sweet potato
PR167	2	–	–	Root	Sweet potato
PR168	2	–	–	Root	Sweet potato

PR169	2	–	–	Root	Sweet potato
PR170	2	–	–	Root	Sweet potato
PR172	2	–	–	Root	Spinach
PR176	2	–	–	Root	Spinach
PR177	2	–	–	Root	Spinach
PR178	2	–	–	Root	Spinach
PR180	3	–	+	Root	Spinach
PR183	2	–	–	Root	Spinach
PR185	1	+	+	Root	Spinach
PR187	3	–	+	Root	Spinach
PR188	2	–	–	Root	Spinach
PR189	2	–	–	Root	Spinach
PR190	2	–	–	Root	Spinach
PR192	2	–	–	Root	Spinach
PR194	2	−	–	Root	Spinach
PR196	3	–	+	Root	Spinach
PR198	2	–	–	Root	Spinach
PR200	3	–	+	Root	Spinach
PR203	3	–	+	Root	Spinach
PR208	3	–	+	Root	Spinach
PR209	3	–	+	Root	Spinach
PR217	2	–	–	Root	Spinach
PR218	2	–	–	Root	Spinach
PR219	2	–	–	Root	Spinach
PR226	2	–	–	Root	Spinach
PR227	2	–	–	Root	Spinach
PR228	2	–	–	Root	Spinach
PR229	2	–	–	Root	Spinach
PR234	2	–	–	Root	Spinach
PR235	2	–	–	Root	Spinach
PR236	3	–	+	Root	Spinach
PR238	2	–	–	Root	Spinach
PR239	3	–	+	Root	Spinach
PR244	2	–	–	Root	Japanese radish
PR245	2	–	–	Root	Japanese radish
PR246	2	–	–	Root	Japanese radish
PR247	2	–	–	Root	Japanese radish
PR254	2	–	–	Root	Spinach
PR256	1	+	+	Root	Spinach
PR257	1	+	+	Root	Spinach
PR258	1	+	+	Root	Spinach
PR259	2	–	–	Root	Spinach

PR262	2	–	–	Root	Spinach
PR263	2	–	–	Root	Spinach
PR265	2	–	–	Root	Spinach
PR266	2	–	–	Root	Spinach
PR267	2	–	–	Root	Spinach
PR268	3	–	+	Root	Spinach
PR269	3	–	+	Root	Spinach
PR273	2	–	–	Root	Spinach
PR276	1	+	+	Root	Spinach
PR277	1	+	+	Root	Spinach
PR279	2	–	–	Root	Spinach
PR280	2	–	–	Root	Spinach
PR282	2	–	–	Root	Spinach
PR283	2	–	–	Root	Spinach
PR284	3	–	+	Root	Spinach
PR285	2	–	–	Root	Spinach
PR286	2	–	–	Root	Spinach
PR287	3	–	+	Root	Spinach
PR289	1	+	+	Root	Spinach
PR293	2	–	–	Root	Spinach
PR294	2	–	–	Root	Spinach
PR295	2	–	–	Root	Spinach
PR305	2	–	–	Root	Leek
PR306	3	–	+	Root	Leek
PR307	2	–	–	Root	Leek
PR308	2	–	–	Root	Leek
PR309	2	–	–	Root	Leek
PR310	2	–	–	Root	Leek
PR311	2	–	–	Root	Spinach
PR312	3	–	+	Root	Spinach
PR315	2	–	–	Root	Spinach
PR316	2	–	–	Root	Spinach
PR318	3	–	+	Root	Spinach
PR327	2	–	–	Root	Spinach
PR328	2	–	–	Root	Spinach
PR336	1	+	+	Root	Spinach
PR337	2	–	–	Root	Spinach
PR338	3	–	+	Root	Spinach
PR343	2	–	–	Root	Spinach
PR344	1	+	+	Root	Spinach
PR345	2	–	–	Root	Spinach
PR346	1	+	+	Root	Spinach

PR347	2	–	–	Root	Spinach
PR348	2	–	–	Root	Spinach
PR349	2	–	–	Root	Spinach
PR350	2	–	–	Root	Spinach
PR353	2	–	–	Root	Spinach
PR354	2	–	–	Root	Spinach
PR355	2	–	–	Root	Spinach
PR356	2	–	–	Root	Spinach
PR359	2	–	–	Root	Spinach
PR363	2	–	–	Root	Spinach
PR364	2	–	–	Root	Spinach
PR365	2	–	–	Root	Spinach
PR374	2	–	–	Root	Carrot
PR384	2	–	–	Root	Spinach
PR385	2	–	–	Root	Spinach
PR386	2	–	–	Root	Spinach
PR387	2	–	–	Root	Spinach
PR388	2	–	–	Root	Spinach
PR392	2	–	–	Root	Spinach
PR394	2	–	–	Root	Spinach
PR395	2	–	–	Root	Spinach
PR404	2	–	–	Root	Leek
PR405	2	–	–	Root	Leek
PR406	2	–	–	Root	Leek
PR416	2	–	–	Root	Spinach
PR417	2	–	–	Root	Spinach
PR419	2	–	–	Root	Spinach
PR426	2	–	–	Root	Leek
PR427	2	–	–	Root	Leek
PR433	2	–	–	Root	Spinach
PR436	1	+	+	Root	Spinach
PR437	3	–	+	Root	Spinach
PR438	2	–	–	Root	Spinach
PR442	2	–	–	Root	Leek
PR445	2	–	–	Root	Leek
PR455	3	–	+	Root	Leek
PR456	2	–	–	Root	Leek
PR463	2	–	–	Root	Japanese mustard spinach
PR464	3	–	+	Root	Japanese mustard spinach
PR467	1	+	+	Root	Japanese mustard spinach
PR468	2	–	–	Root	Japanese mustard spinach
PR476	2	–	–	Root	Carrot

PR493	3	–	+	Root	Spinach
PR494	2	–	–	Root	Spinach
PR495	2	–	–	Root	Spinach
PR505	3	–	+	Root	Spinach
PR506	3	–	+	Root	Spinach
PR507	3	–	+	Root	Spinach
PR515	2	–	–	Root	Spinach
PR516	2	–	–	Root	Spinach
PR523	2	–	–	Root	Leek
PR524	3	–	+	Root	Leek
PR525	2	–	–	Root	Leek
PR526	2	–	–	Root	Leek
PR528	2	–	–	Root	Leek

Table S3. Summary of assembled and annotated whole-genome sequences of nine *Priestia megaterium* strains.

Strain	AHL degradation group	DDBJ/ENA/GenBank accession numbers	DRA accession number	Total length (bp)	Contigs	G+C Content (%)	N50	CDSs	rRNA genes	tRNA genes
S70	1	BAAGDF010000001 –BAAGDF010000117	DRR623159	5,453,662	117	37.9	833,747	5,587	6	132
PL125	1	BAAGDG010000001 –BAAGDG010000051	DRR623160	5,311,346	51	38.0	1,208,942	5,415	4	121
PR185	1	BAAGDH010000001 –BAAGDH010000057	DRR623161	5,334,896	57	37.9	1,180,664	5,528	6	123
S67	2	BAAGDI010000001 –BAAGDI010000106	DRR623162	6,167,012	106	37.4	1,203,775	6,362	6	122
PL128	2	BAAGDJ010000001 –BAAGDJ010000088	DRR623163	6,139,504	88	37.4	1,227,571	6,345	5	130
PR54	2	BAAGDK010000001 –BAAGDK010000096	DRR623164	6,048,516	96	37.4	1,278,049	6,252	6	129
S228	3	BAAGDL010000001 –BAAGDL010000065	DRR623165	5,505,562	65	37.8	1,186,300	5,625	5	110
PL103	3	BAAGDM010000001 –BAAGDM010000076	DRR623166	5,587,962	76	37.7	769,032	5,801	6	123
PR236	3	BAAGDN010000001 –BAAGDN010000083	DRR623167	5,826,667	83	37.5	1,043,830	6,055	7	136
